# Research trends in esketamine for depression over the past decade: a bibliometric analysis

**DOI:** 10.3389/fpsyt.2025.1621830

**Published:** 2025-06-25

**Authors:** Baozhou Zhang, Yifan Liu, Jinwei Zheng, Junping Chen

**Affiliations:** ^1^ Department of Anesthesiology, Ningbo No.2 Hospital, Ningbo, Zhejiang, China; ^2^ School of Medicine, Shaoxing University, Shaoxing, Zhejiang, China

**Keywords:** research trend, bibliometric analysis, esketamine, depression, antidepressant

## Abstract

**Background:**

Patients suffering from depression frequently encounter extended periods of low moods and lack of enjoyment or enthusiasm for activities. It leads to suicidal thoughts and presents a potential hazard to their safety. Nowadays, there has been significant progress in researching the effectiveness and safety of esketamine in treating depression. Hence, this paper employs bibliometric analysis to investigate the evolution and future research trajectories of this domain.

**Methods:**

We utilize Excel, VOSviewer, and CiteSpace software to generate bibliometric network visualizations to analyze, construct, and quantitatively evaluate pertinent literature, which facilitates a lucid and intuitive presentation of the trends and frontiers in this research domain.

**Results:**

Annual publications increased from 2015 to 2024, totaling 925 articles, with 286 studies published in 2024. The USA published the most papers (n=308), followed by China (n=260) and Canada (n=114). Three of the top journals were Journal of Affective Disorders (n=56,IF=4.90), Frontiers in Psychiatry (n=38,IF=5.44), and International Journal of Neuropsychopharmacology (n=21,IF=4.50). The most published authors were McIntyre, Roger S (n=52), followed by Hashimoto, Kenji (n=49), Rosenblat, Joshua D (n=41). The keywords that have been relevant to the topic for the last decade are “treatment-resistant depression”, “efficacy”, “antidepressant” and “suicidal ideation”.

**Conclusions:**

This bibliometric analysis showed a significant increase in research on the use of esketamine in the treatment of depression. The main focus of current research is still the assessment of long-term use safety. In addition, the huge difference in research resources between developed countries and low- and middle-income countries remains an unresolved issue.

## 1 Introduction

Depression has become the leading cause of disability worldwide (1). World Health Organization data reveal that one person commits suicide every 40 seconds, with 77% of these suicides occurring in low- and middle-income countries. Since the pandemic began, the incidences of major depressive disorder and anxiety disorders have risen by 28% and 26%, respectively, adding 53 million new cases (2). About one-third of patients do not respond adequately to existing medication and psychotherapy treatments (3). Consequently, there is a pressing need to develop new strategies to treat major depression, particularly treatment-resistant depression (TRD) (4).

Among the various treatment options, esketamine, an N-methyl-d-aspartate receptor antagonist found in the S-enantiomer of ketamine racemate, has shown potential effectiveness in treating TRD (5–8). Esketamine provides quick relief from depressive symptoms, often within hours of use. This fast action is crucial for those experiencing severe symptoms or suicidal thoughts (9). Unlike traditional antidepressants that target neurotransmitters like serotonin, esketamine increases glutamate levels, a major neurotransmitter that enhances brain function and synaptic connections. This can improve mood and cognitive abilities (10). Additionally, studies suggest that esketamine stimulates the formation of new neural pathways, potentially reversing the effects of chronic stress and depression (11). However, it’s important to note the potential side effects of esketamine use, including ulcerative cystitis, cognitive impairment, and addiction risk (12–15). Nevertheless, esketamine offers a powerful option for managing severe and resistant forms of depression, providing hope for those who have not benefited from conventional treatments (16).

Despite the extensive clinical studies conducted on esketamine, there remains a notable absence of systematic bibliometric analyses aimed at mapping global research trends. Previous reviews have predominantly concentrated on clinical research, yet they have not provided quantitative insights into the publishing model (17). Furthermore, the regional disparities in TRD research underscore the need for a comprehensive global bibliometric perspective (18).

Bibliometric analysis has become a key tool for visualizing high-impact research and identifying trends in scientific literature (19). It helps analyze article and journal performance, cooperation models, research elements, and the knowledge structure of specific fields (20, 21). Our study focused on examining the use of esketamine in depression treatment over the past decade, using network diagrams to explore interactions among nations, educational institutions, journals, authors, and keywords. While previous bibliometric analyses have examined the research landscape of ketamine and esketamine in the context of depression, this study specifically concentrates on the post-approval period (2015-2024) (22–24). It aims to elucidate research trends following the United States Food and Drug Administration’s approval of esketamine for the treatment of treatment-resistant depression (TRD) in 2019—a phase that is underrepresented in earlier reviews. This study is more timely and contains 286 articles in 2024, which may affect the results of bibliometrics.

## 2 Materials and methods

### 2.1 Strategy for search

**Figure 1:**
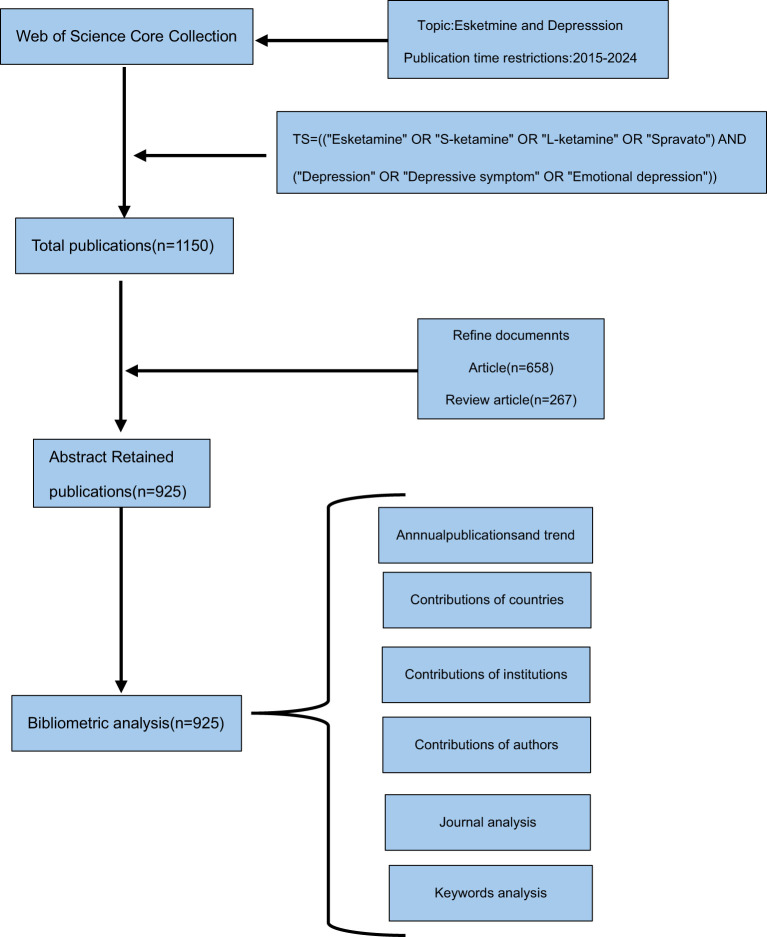
This study was based on the publication flowchart above.

The Science Citation Index Expanded and the Social Sciences Citation Index within the Web of Science Core Collection(WoSCC) were accessed online on December 31, 2024. The temporal scope of the study spans from 2015 to 2024. The search strategy employed is defined by the following terms: TS=((“Esketamine” OR “S-ketamine” OR “L-ketamine” OR “Spravato”) AND (“Depression” OR “Depressive symptom” OR “Emotional depression”)). Without any language limitations, we acquired all the information within a 24-hour period on December 31, 2024. A grand total of 1150 studies were acquired. The search flow detail was illustrated in **Figure 1**. Two independent researchers performed a screening of the titles and abstracts, excluding literature types considered to be of lesser significance, such as editorial materials, letters, early access publications, corrections, news items, retracted publications, and book chapters. Any disagreements were resolved through discussion or consultation with the corresponding author. This process resulted in the identification of 925 articles pertinent to our research, comprising 658 original research articles and 267 review articles.

### 2.2 Statistical analysis

Microsoft Office Excel 2020, developed in Redmond, WA, USA, is used to analyze Web of Science collection data. To demonstrate the correlations among publications, we utilized Excel 2020 to generate graphs. With VOSviewer (1.6.19.0), it can view three types of visual maps: network visualization, overlap visualization, and density visualization (25). VOSviewer represents countries, institutions, journals, and authors as nodes, depending on their frequency of occurrence in the title and abstract of papers analyzed (26). The CiteSpace software (6.1.6.0) enables the visualization of emerging patterns and sudden shifts, as well (27). The analysis conducted by CiteSpace spans from 2015 to 2024, with each interval representing a year. During each iteration, a single type of node is chosen, and the selection process involves using a g index (k=25) and a minimum duration (MD=1).

## 3 Results

### 3.1 Trends in annual publication growth

**Figure 2:**
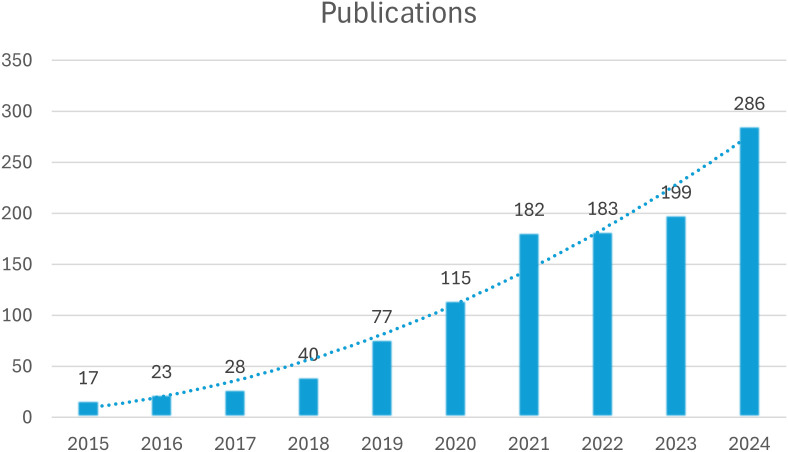
Trend chart of the number of publications in the past decade.

**Figure 2** shows the evolution trend of the number of published papers on the treatment of depression with esketamine in the past ten years. The data showed that the research in this field showed an obvious upward trend: in the accumulation stage from 2015 to 2020, the annual number of publications increased steadily from 17 to 115, showing the continuous attention of the academic community to this therapy. Annual publications surged from 115 (2020) to 286 (2024) post-FDA approval, reflecting intensified research focus.

### 3.2 Countries and institutions with high productivity

**Figure 3:**
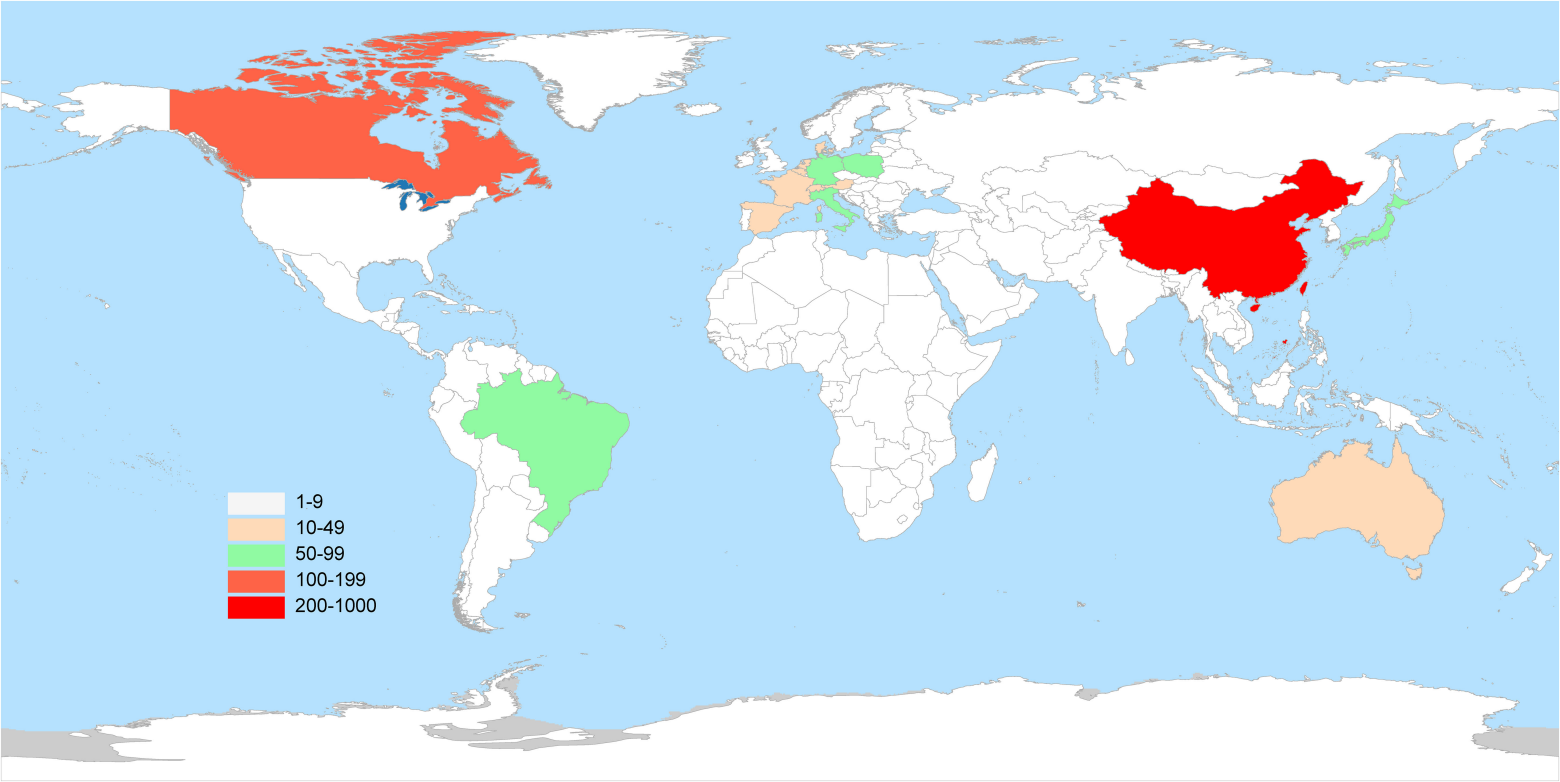
Map of national contributions based on total publications.

**Figure 4:**
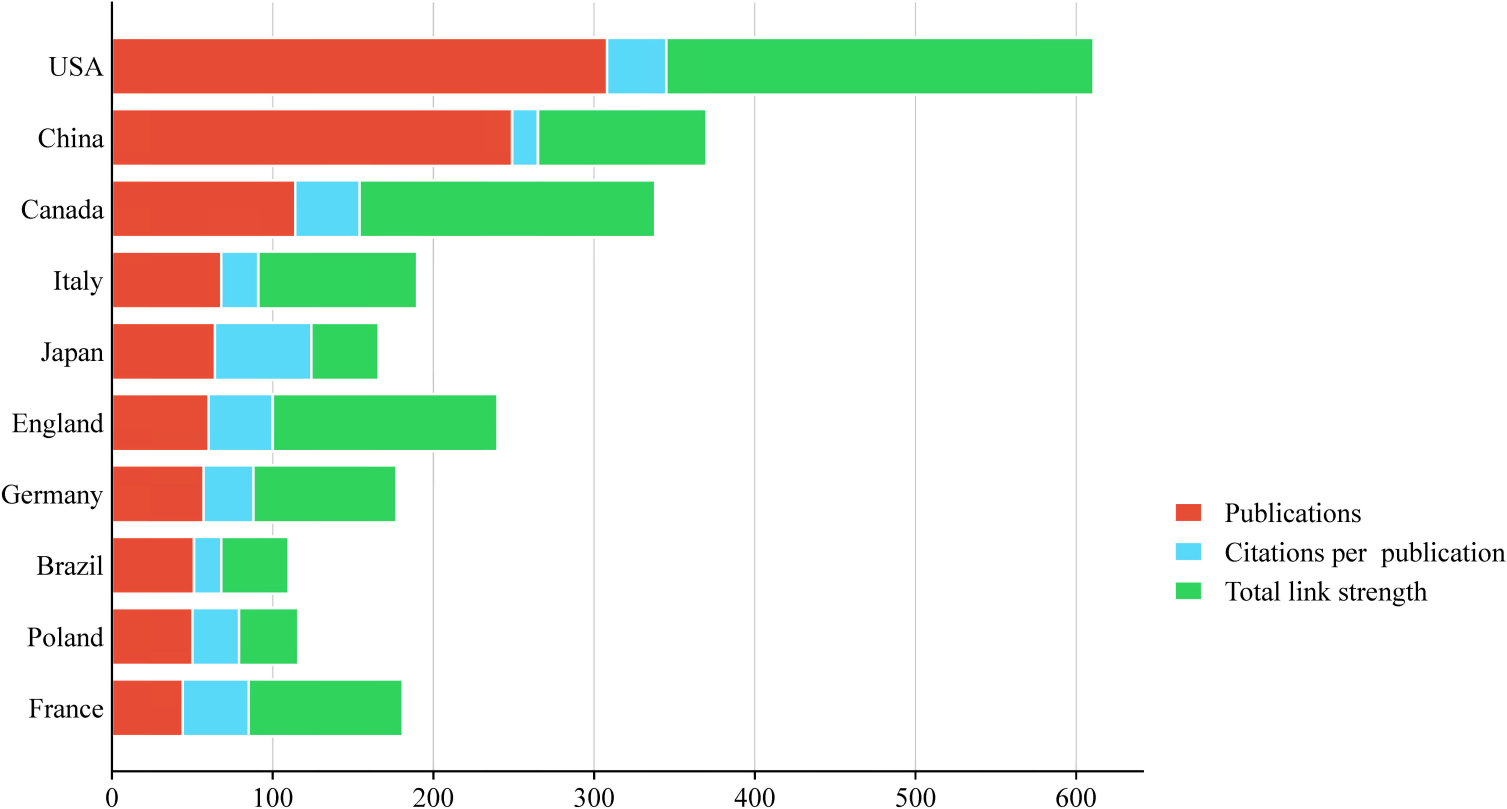
Top 10 countries with the largest number of publications.

The literature analysis of esketamine in this study covered 50 countries around the world. The visualized distribution map of geographical contribution shown in **Figure 3** shows that the United States and China are the most important research output countries in this field. Further through the global academic influence ranking revealed by **Figure 4**, it can be seen that among the top 10 countries, the United States dominates with 308 articles (accounting for nearly 30%), and China ranks second with 24% of global publications. The predominance of United States publications is likely indicative of Janssen Pharmaceuticals’ financial support for esketamine trials (McIntyre et al., 2021). Concurrently, the increasing contributions from China correspond with the nation’s ‘Healthy China 2030’ initiative, which emphasizes the prioritization of innovation in mental health. It is worth noting that the United States is particularly prominent in terms of total citations, with a total of 11,374 times, far more than other countries. In terms of citation quality, Japan’s average citation rate ranked first with 51.00%. The map uses a three-color indicator system: the red column indicates the number of national publications, the blue column reflects the number of citations per article, and the green column represents the Total Link Strength—a bibliometric core indicator that reflects academic influence through the strength of the cooperative network, and its value is positively correlated with the status of the field.

**Figure 5:**
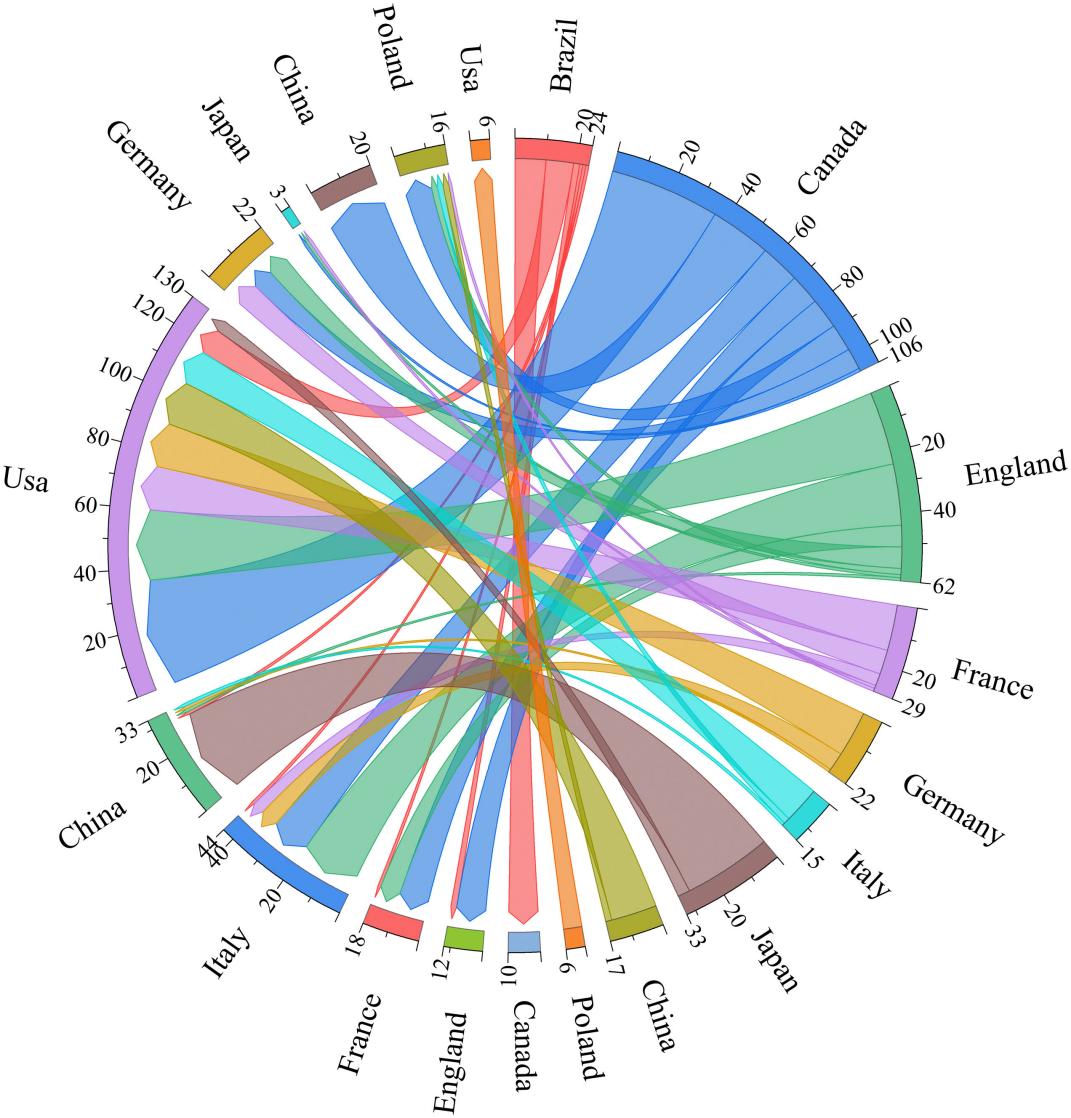
The cooperation chord diagram between the top 10 countries.

Chord diagram was used to visualize each country’s contributions in esketamine for depression, as shown in **Figure 5**. The National Contribution Atlas consists of 10 nodes, which show case the cooperative research networks formed in 10 countries or regions focusing on esketamine for the treatment of depression. The number of papers published in each country (n≥44) is represented by the length of the circle in the chord graph. Furthermore, the width of the line signifies the intensity of the connection between two nations or areas. The analysis of inter-country cooperation shows that the USA is the main center of the network and is involved in regular cooperation with Canada. The importance of global collaboration in progressing research on esketamine for depression treatment is emphasized by this discovery.

**Figure 6:**
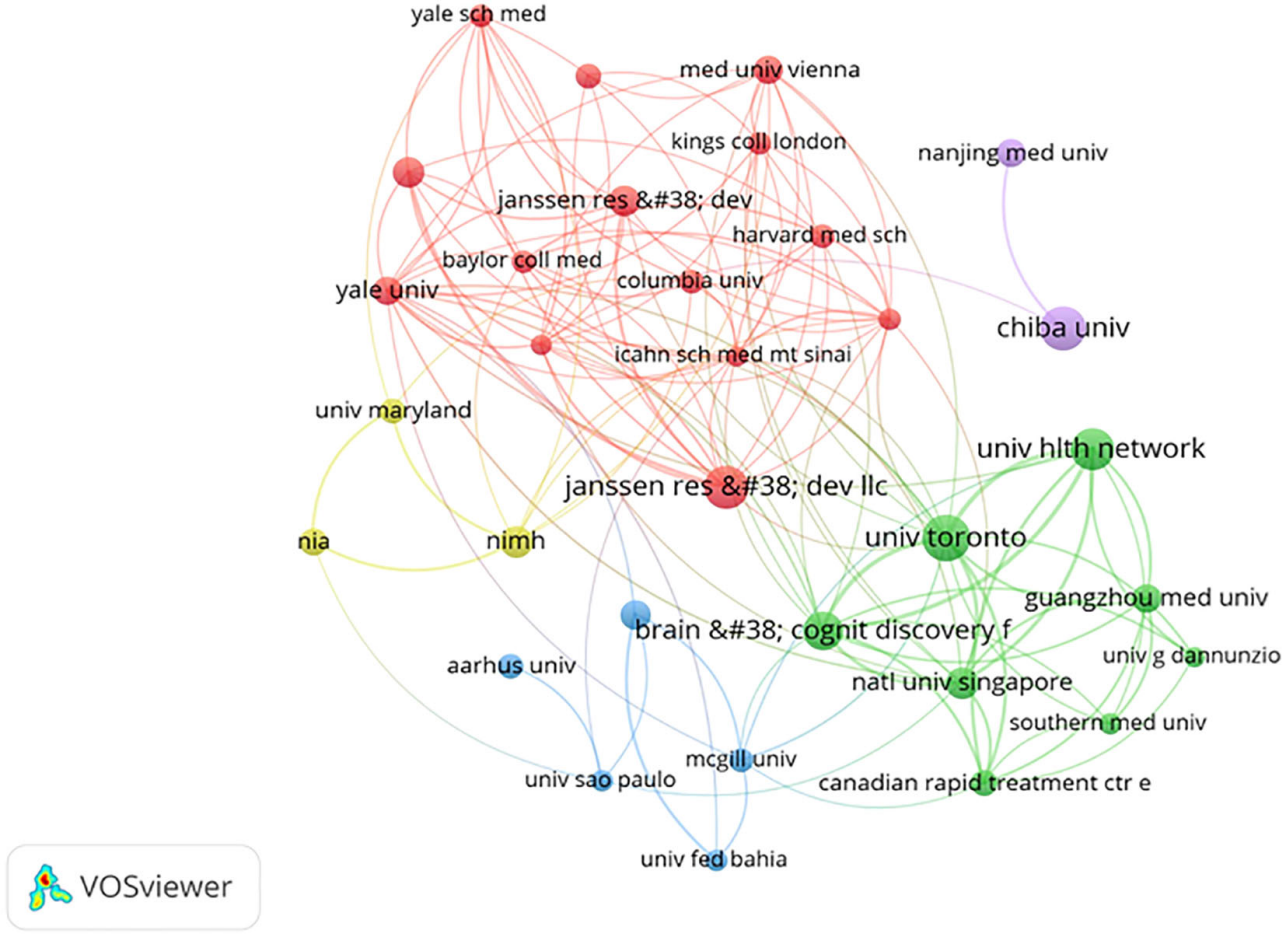
Institutional cooperation clustering map.

**Figure 6** shows the results of our co-authored analysis on VOSviewer, and found that 33 institutions have made significant contributions to the study of esketamine in the treatment of depression. The size of the circle in the diagram is positively correlated with the number of publications. The thicker the line, the closer the cooperation between each other. **Table 1** provides details on the leading 10 institutions, which include University of Toronto (n=62), University Health Network (n=47), Brain & Cognitive Discovery Foundation (n=44). The majority of these organizations are situated in the USA and Canada. It is worth emphasizing that despite publishing only 23 related articles, Janssen Research & Development has amassed an impressive 2452 citations. This indicates that the papers are of excellent quality. **Table 1** reveals that the three institutions affiliated with Janssen have published a total of 91 related articles, thereby dominating the body of literature. An analysis of the funding sources for these articles indicates a strong connection to the institutions’ sponsorship of researchers (28–30). This observation, however, prompts concerns regarding the potential influence on the research agenda, particularly in the study of esketamine. While collaboration with industry partners can expedite scientific advancements, it is noteworthy that research focusing on long-term safety constitutes only 18% of the publications. This disparity underscores the necessity for enhanced regulatory oversight prior to the commercialization of pharmaceutical products.

**Table 1:** The top ten institutions that are actively treating depression using esketamine.

Rank	Institution	Document	Citations	Citation Per Document
1	University of Toronto	62	1800	29
2	University Health Network	47	1171	25
3	Brain & Cognitive Discovery Foundation	44	1201	27
4	Janssen Research & Development, LLC	44	2451	56
5	National University of Singapore	28	889	32
6	National Institute of Mental Health	26	2375	91
7	Janssen Scientific Affairs, LLC	24	251	10
8	Janssen Research & Development	23	2452	107
9	Yale University	23	1283	56
10	Universidade Federal de São Paulo	22	606	28

### 3.3 Journal analysis

**Figure 7:**
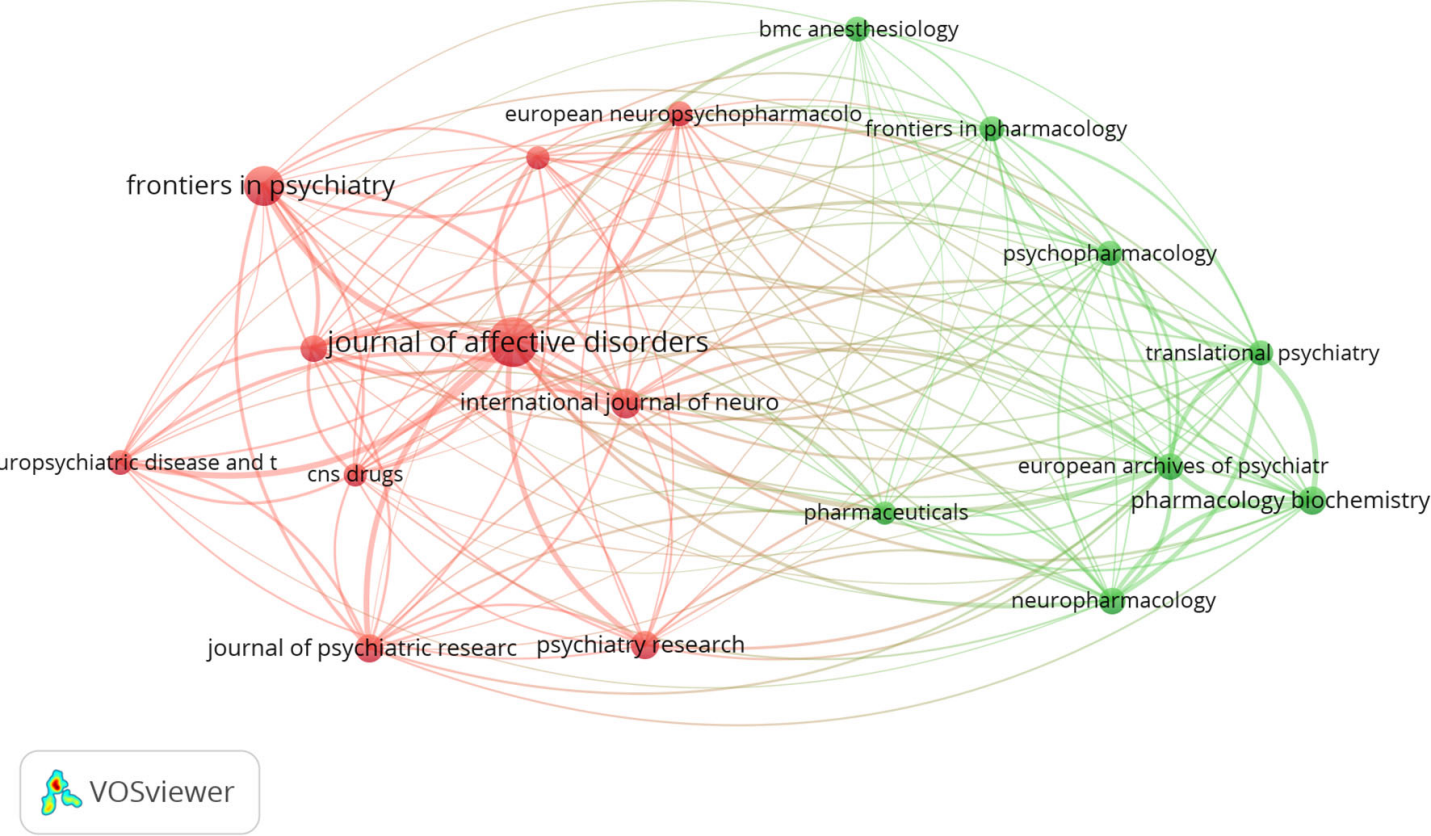
Journal cooperation map.

We utilized VOSviewer to identify the most productive and widely cited journals in the field of esketamine for depression. **Table 2** lists the top 10 journals with the highest activity and co-citation frequency. The top 3 productive journals were Journal of Affective Disorders (n=56, IF=4.90), Frontiers in Psychiatry (n=38, IF=5.44), and International Journal of Neuropsychopharmacology (n=21, IF=4.50) among the mentioned publications. It is noteworthy that these three journals published 49.14% of the total number of articles published, which can be considered as the core journals in the field. Journal of Clinical Psychiatry published only 16 articles and collected 614 citations, which indicates that the quality of articles in this journal is high. **Figure 7** shows the network of collaborative relationships between journals generated by Vosviewer, with thicker lines indicating that the journals collaborate more closely with each other and that the probability of citation between articles published by the journals is higher.

**Table 2:** Esketamine therapy for depression Journals and co-cited journals related to.

Rank	Journals	Country	Publications	IF (2024)	Citations	JCR
1	J Affect Disord	Netherlands	56	4.90	1218	Q1
2	Front Psychiatry	Switzerland	38	5.44	206	Q2
3	Int J Neuropsychopharmacol	United Kingdom	21	4.50	728	Q1
4	J Psychiatr Res	United Kingdom	20	3.70	391	Q2
5	Pharmacol Biochem Behav	United States	18	3.30	515	Q1
6	Psychiatry Res	Ireland	18	4.23	276	Q1
7	Eur Arch Psychiatry Clin Neurosci	Germany	16	3.50	480	Q1
8	J Clin Psychiatry	United States	16	4.50	614	Q1
9	Neuropharmacology	United Kingdom	16	4.60	286	Q1
10	BMC Anesthesiol	United Kingdom	15	2.30	127	Q3

### 3.4 Author analysis

**Figure 8:**
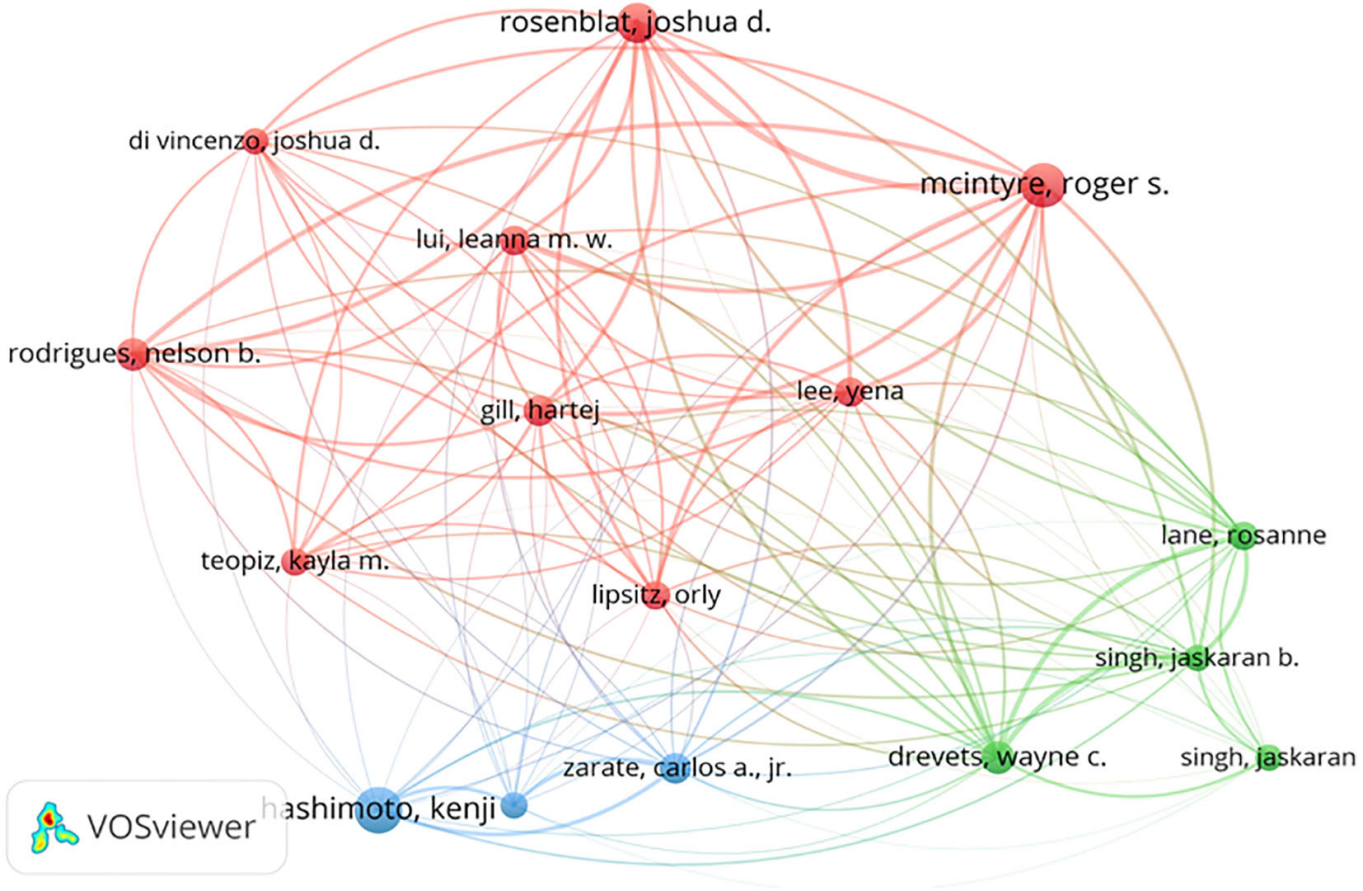
Author cooperative clustering map.

In **Table 3**, the top ten authors and their co-citations are listed who have made the most contributions in researching esketamine as a treatment for depression among all the scholars involved in the study. An intuitive observation reveals that McIntyre, Roger S (n=52) holds the record for publishing the highest number of articles in this field, closely followed by Hashimoto, Kenji (n=49) and Rosenblat, Joshua D (n=41). Nevertheless, it is crucial to emphasize the specific influence of Zarate, Carlos A., Jr, who despite publishing only 19 pertinent papers, has managed to accumulate an astounding 2667 citations. It is clear from this that Zarate, CarlosA., Jr 's publications are of very high quality and are valuable as references. These findings are consistent with the H index. Authors with at least 15 publications are shown in **Figure 8**. The size of the circle in the figure represents the number of papers published by the author, and the number of lines represents the intensity of cooperation between the authors.

**Table 3:** Esketamine for the treatment of depression: Active authors and co-cited authors in the top 10.

Rank	Author	Publications	Citations	Citation Per Publication	H-index
1	McIntyre, Roger S	52	1559	30	23
2	Hashimoto, Kenji	49	3125	64	31
3	Rosenblat, Joshua D	41	1301	32	22
4	Drevets, Wayne C	23	3147	137	19
5	Teopiz, Kayla M	23	455	20	13
6	Rodrigues, Nelson B	22	752	34	16
7	Gill, Hartej	20	715	36	15
8	Mansur, Rodrigo B	19	797	42	15
9	Zarate, Carlos A., Jr	19	2667	140	22
10	Gould, Todd D	18	1762	98	15

### 3.5 Documents analysis

**Figure 9:**
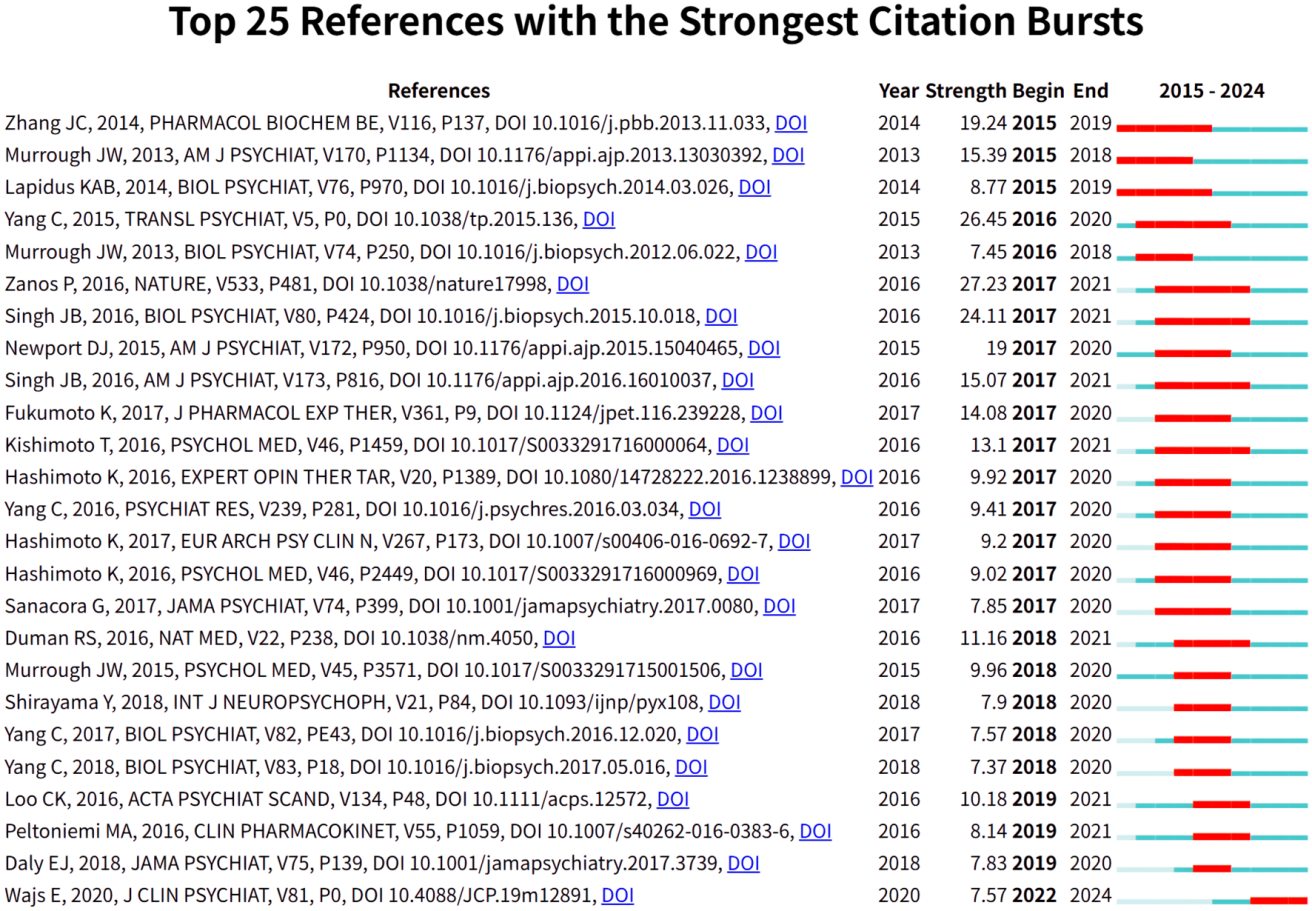
Citation bursts associated with esketamine in depression treatment: top 25 references.

To gain insight into the progress of esketamine as a treatment for depression, a reference analysis was conducted during the research. CiteSpace was utilized to visualize the highlighting time of cited articles after studying the literature that was cited most frequently (**Figure 9**). It is worth noting that most of the related articles were about investigating the efficacy and safety of esketamine in the treatment of depression. In addition, Ewa Wajs’s article on The Journal of Clinical Psychiatry, published in 2020, is still influential in recent years.

### 3.6 Keywords analysis

**Figure 10:**
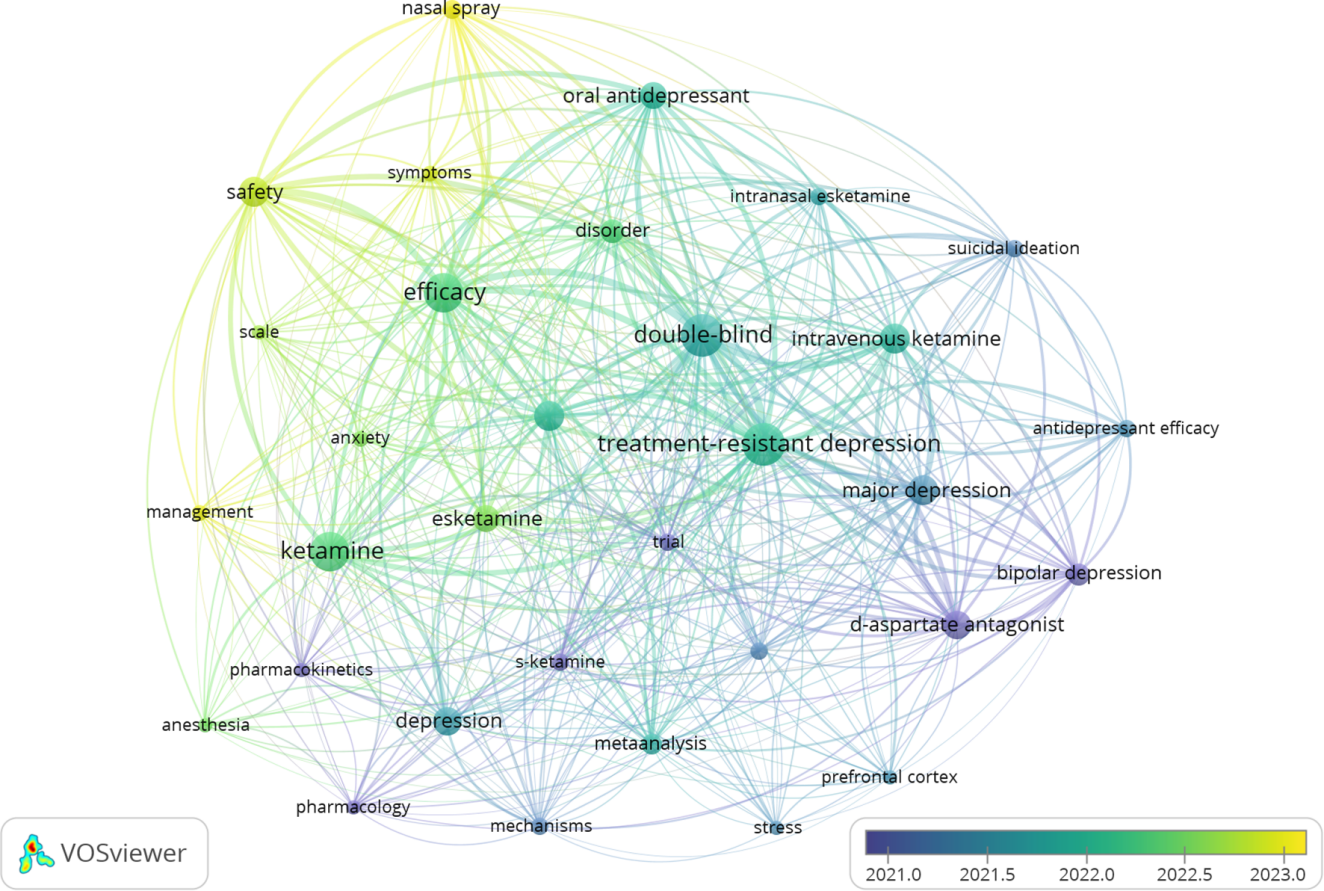
Keywords change over time clustering map.

Examining the co-occurrence of keywords in this field can reveal research subjects and directions. VOSviewer was used to extract 10 keywords. According to **Table 4**, the top 10 keywords have a frequency of over 36 occurrences. The keywords that appeared most frequently include “Treatment-Resistant Depression” (n=364), “Efficacy” (n =232), “Antidepressant” (n=187), “Suicidal Ideation” (n=74), and “Safety” (n=153). These keywords represent the main theme of esketamine in the treatment of depression. Centrality analysis shows that Treatment-Resistant Depression (0.24) is the main focus, with Esketamine (0.18) as the key research subject, particularly for its role in quickly reducing suicidal thoughts (0.17). The study compares traditional antidepressants (0.13) and different delivery methods (Nasal Spray and Intravenous Ketamine, both 0.05), emphasizing efficacy (0.06) and safety (0.07). To provide a clear depiction of frequently occurring keywords, we constructed a keyword network diagram (**Figure 10**) using VOSviewer, illustrating the temporal evolution of research topics. In this diagram, the color yellow denotes recent research hotspots, with “safety” emerging as the most prominent keyword. Following the approval of Spravato in 2019, global regulatory bodies mandated its restricted use within medical institutions, owing to concerns regarding its potential for abuse and associated post-medication risks. Consequently, the issuance of the FDA black box warning in 2020 led to an 83% increase in the occurrence of safety-related keywords, rising from 47 instances in 2020 to 86 instances in 2021. The transition in keyword emphasis from “rapid antidepressant effect” prior to 2019 to “safety” post-2020 underscores the clinical discourse surrounding the assessment of risks versus benefits. This shift contrasts with industry disclosures that predominantly highlight efficacy, thereby underscoring the imperative for developing a robust pharmacovigilance framework.

**Table 4:** Esketamine’s top 10 keywords for depression treatment.

Rank	Keywords	Occurrences	Total link strength	Centrality
1	Esketamine	467	797	0.18
2	Treatment-Resistant Depression	364	755	0.24
3	Efficacy	232	569	0.06
4	Antidepressant	187	374	0.13
5	Safety	153	398	0.07
6	Intravenous Ketamine	130	300	0.05
7	Major Depressive Disorder	120	256	0.11
8	Suicidal Ideation	74	200	0.17
9	Nasal Spray	51	179	0.05
10	Suicide	36	98	0.08

## 4 Discussion

This study has conducted a comprehensive bibliometric analysis to assess the application and research trends of esketamine in the treatment of depression over the past decade. Our findings not only reveal the global research dynamics in this field but also highlight the significant contributions of specific regions and research institutions.

### 4.1 Global research dynamics and collaborative gaps

Since the US Food and Drug Administration (FDA) approved esketamine for TRD, the explosive growth of related research has highlighted the great potential of the drug to cope with TRD (31–33). The scientific research output of the United States and China dominates, which is closely related to the large investment in psychiatric innovation and infrastructure in the two countries. North America and Europe lead in citation influence, but 77% of suicides worldwide occur in low- and middle-income countries (LMICs), and research in these regions is seriously underrepresented. These countries typically encounter challenges including inadequate medical infrastructure, delayed diagnosis, elevated treatment costs, cultural belief disparities, and restricted research funding (34). These issues have significantly impacted patient treatment outcomes and survival rates. This imbalance highlights the urgency of promoting fair global collaboration. Initiatives such as cross-border funding projects, data-sharing platforms, and capacity-building projects can bridge this gap and ensure that the benefits of esketamine benefit the people most affected by TRD.

### 4.2 Author and institutional contributions

The significant contributions of scholars like McIntyre and Hashimoto, alongside the exemplary performance of institutions such as the University of Toronto, underscore the pivotal role of interdisciplinary collaboration in advancing the study of esketamine. Notably, Zarate’s research, although limited in quantity, has exerted a disproportionately substantial influence, thereby exemplifying the academic principle of valuing quality over quantity.

### 4.3 Journal contributions

Core journals like the Journal of Affective Disorders and Frontiers in Psychiatry have been instrumental in disseminating high-impact research. However, the concentration of publications in Q1/Q2 journals may inadvertently exclude novel studies from LMICs, where open-access barriers persist. Encouraging hybrid publishing models and waivers for researchers in resource-limited settings could democratize knowledge exchange.

### 4.4 Theme changes

An efficient method of assessing the degree of relation between articles is citation analysis (35). From these high-quality citation analyses, the comparative analysis of different years highlights how research has shifted to optimizing treatment options and minimizing adverse effects (36). For example, the research topic has shifted from the early “rapid antidepressant effect” to the current “safety” and “suicidal ideation”. This evolution echoes the clinical reality: although the rapid onset of esketamine is revolutionary, its long-term risks (such as dissociation symptoms, cognitive impairment, and dependence risks) still need to be rigorously assessed (37). This shift emphasizes the maturity of the field, aiming to balance efficacy and safety and ensure that the benefits of esketamine treatment continue over time without compromising patient health (38).

### 4.5 Future research directions

Future studies will likely explore combination therapies that enhance esketamine’s effects or mitigate its side effects. Additionally, identifying patient subgroups that benefit most from esketamine could tailor treatments more effectively, ensuring that those most likely to benefit receive the therapy.

In conclusion, significant advancements have been made in the investigation of esketamine as a solution to the pressing demand for effective treatments for treatment-resistant depression. While ongoing research and emerging data are anticipated to enhance its clinical application, evidence regarding its long-term safety remains insufficient. Future studies need to address this deficiency to facilitate the formulation of definitive clinical guidelines. This progress underscores the dynamic nature of psychiatric treatment research and highlights the necessity for continuous investigation to fully realize the therapeutic potential of esketamine.

### 4.6 Limitations

This study has limitations, such as relying on WoSCC as the primary data source, potentially excluding other databases and recent low-cited literature, which may affect result comprehensiveness. Language bias might overlook non-English studies from LMICs, where antidepressant resistance is higher. Citation bias could favor prestigious institutions and overrate industry-funded research. Excluding grey literature, like clinical trial registries, limits access to unpublished negative results. Despite these issues, the study offers valuable insights into esketamine treatment for depression and supports future research.

## 5 Conclusion

This bibliometric analysis reveals a significant surge in scholarly investigations concerning the treatment of depression using esketamine. Despite the publication of 286 pertinent articles in 2024, the predominant focus of research remains on the safety associated with long-term use. Furthermore, the substantial disparity in research resources between developed nations and low- and middle-income countries persists unresolved.
